# Pb(II) Uptake from Polluted Irrigation Water Using Anatase TiO_2_ Nanoadsorbent

**DOI:** 10.3390/molecules28124596

**Published:** 2023-06-07

**Authors:** Miguel A. Vasquez-Caballero, Yamerson Canchanya-Huaman, Angie F. Mayta-Armas, Jemina Pomalaya-Velasco, Noemi-Raquel Checca-Huaman, Yéssica Bendezú-Roca, Juan A. Ramos-Guivar

**Affiliations:** 1 Laboratorio de No Metálicos, Facultad de Ingeniería Química, Universidad Nacional del Centro del Perú (UNCP), Av. Mariscal Ramón Castilla Nº 3909, El Tambo, Huancayo 12000, Peruyessiben@yahoo.com (Y.B.-R.); 2 Centro Brasileiro de Pesquisas Físicas, Rio de Janeiro 22290-180, RJ, Brazil; 3 Grupo de Investigación de Nanotecnología Aplicada para Biorremediación Ambiental, Energía, Biomedicina y Agricultura (NANOTECH), Facultad de Ciencias Físicas, Universidad Nacional Mayor de San Marcos, Av. Venezuela Cdra 34 S/N, Ciudad Universitaria, Lima 15081, Peru

**Keywords:** water bodies recuperation, adsorbents post-exposure properties, anatase–lead complexation, water cleaning strategies

## Abstract

The adsorption characteristics of titanium dioxide nanoparticles (nano-TiO_2_) for the removal of Pb(II) from irrigation water were investigated in this work. To accomplish this, several adsorption factors, such as contact time and pH, were tested to assess adsorption efficiencies and mechanisms. Before and after the adsorption experiments, commercial nano-TiO_2_ was studied using X-ray diffraction (XRD), scanning and transmission electron microscopy (SEM and TEM), energy dispersive spectroscopy (EDS), and X-ray photoelectron spectroscopy (XPS). The outcomes showed that anatase nano-TiO_2_ was remarkably efficient in cleaning Pb(II) from water, with a removal efficiency of more than 99% after only one hour of contact time at a pH of 6.5. Adsorption isotherms and kinetic adsorption data matched the Langmuir and Sips models quite well, showing that the adsorption process occurred at homogenous sites on the surface of nano-TiO_2_ by forming a Pb(II) adsorbate monolayer. The XRD and TEM analysis of nano-TiO_2_ following the adsorption procedure revealed a non-affected single phase (anatase) with crystallite sizes of 9.9 nm and particle sizes of 22.46 nm, respectively. According to the XPS data and analyzed adsorption data, Pb ions accumulated on the surface of nano-TiO_2_ through a three-step mechanism involving ion exchange and hydrogen bonding mechanisms. Overall, the findings indicate that nano-TiO_2_ has the potential to be used as an effective and long-lasting mesoporous adsorbent in the treatment and cleaning of Pb(II) from water bodies.

## 1. Introduction

Pollution of the environment with heavy metals has become a big issue across the world, affecting both industrialized and developing nations [1]. Water pollution and scarcity are the two most serious environmental issues threatening the existence of plants, animals, and people in the twenty-first century. Human activities have disrupted the natural recycling of water and its purifying mechanisms, resulting in a shortage of water for human use [2]. According to the 2018 United Nations Global Water Development Report, industrial and household water consumption will grow far faster than agricultural need, despite agriculture remaining the largest overall consumer [3]. Water is now utilized for agriculture 70% of the time, with irrigation being the most common application. Electroplating, metallurgy, steel, fertilizers, pesticides, textiles, paints, glass, synthetic materials, and mining are among the sectors that discharge heavy-metal-containing wastewater into the environment [4,5]. Even at low concentrations, these pollutants are very hazardous to human health; thus, they must be treated before disposal [6].

Lead, Pb(II), is one of the most damaging heavy metals; its influence on water contamination has sparked international concern [7]. Both natural weathering processes and human-made operations, such as mining, making batteries, and electroplating, can release Pb(II) into the environment [8]. Because of its bioaccumulation, toxicity, and persistence, Pb(II) is an extremely harmful chemical [9,10]. Infants, children under the age of six, the fetus, and pregnant women are the most vulnerable to poor health impacts [11]. The effects on the central nervous system can be especially severe, including cancer, anemia, and damage to the brain and other organ systems [12,13]. The World Health Organization (WHO) suggests keeping the amount of lead in drinking water to a maximum of 0.01 mg L^−1^ [11].

Several methods have been developed to reduce the levels of Pb(II) and other heavy metals in polluted water, including adsorption, biosorption, chemical precipitation, ion exchange, membrane separation, ion treatment, electrochemical treatment, recovery by evaporation, redox precipitation, and filtration [12,14,15,16]. Adsorption has numerous benefits over the other approaches [17,18]. It is easy to use and there are a wide range of adsorbents that can be reused after the desorption process in various adsorption cycles, allowing for the reasonably priced separation of pollutants [19]. Nowadays, the utilization of nanoparticles (NPs) as an absorbent for metal pollutants is a highly promising option [20]. Nanomaterials offer excellent characteristics for reducing pollutants in water. Due to their small size [21,22] and large surface area, NPs easily disperse in liquids where they can interact with a variety of chemical species [19].

Nanostructures have emerged as a very attractive alternative for water cleanup due to their huge surface-to-volume ratios, which are excellent for surface reactions [23]. Numerous studies have demonstrated that heavy metals, such as Pb(II) and Cd(II), can be effectively removed from aqueous solutions using nano-TiO_2_. As a result, investigations on the use of nano-TiO_2_ in wastewater treatment are extremely beneficial to the aquatic environment [24]. Moreover, numerous TiO_2_-based nanocomposites have been investigated to increase these materials’ adsorption capacity [25,26]. TiO_2_ polymorphs include anatase, rutile, and brookite, which are the most prevalent. Each of these polymorphs has unique structural, physical, and chemical features that influence their ability for heavy metal adsorption. Anatase, for example, has a bigger surface area and porosity than rutile and brookite, allowing it to adsorb more metals. Yet, under specific pH and temperature circumstances, rutile and brookite are more stable than anatase [27,28]. Despite their remarkable Pb(II) removal properties in simulated aliquots, little literature is available concerning nano-TiO_2_ adsorption performance in real waters. Certainly, diverse nano-TiO_2_ polymorphs will exhibit different removal properties in river, lake, and sea samples containing heavy metals, and, more importantly, the conservation of the physicochemical properties of nano-TiO_2_ will guarantee their environmental sustainability and reuse.

In that sense, the pollution of water bodies by Pb(II) is a current concern—taking in and getting rid of this metal is important and an interesting challenge. This work used nano-TiO_2_ as an alternative to remove Pb(II) from irrigation water with a corrected starting concentration of 47.54 mg L^−1^, and its removal effectiveness was evaluated. The water samples were collected from the CIMIRM irrigation canal in Peru’s province of Jauja. Analytic techniques were used to evaluate nano-TiO_2_ before and after Pb adsorption—this last to ensure their physicochemical and reusability properties. Multiparametric adsorption analysis, including adsorption kinetics, the effect of pH on adsorption, adsorbent dose on adsorption capacity, and the behavior of experimental data in relation to adsorption isotherms, were also investigated. The results indicate that anatase nano-TiO_2_ is an alternative solution to the Pb(II) water cleaning problem, including modified surface water samples that exceed the permissible levels needed for agricultural irrigation, e.g., [29].

## 2. Results and Discussions

### 2.1. Characterization of Nano-TiO_2_

#### 2.1.1. XRD and Rietveld Analysis

**Figure 1:**
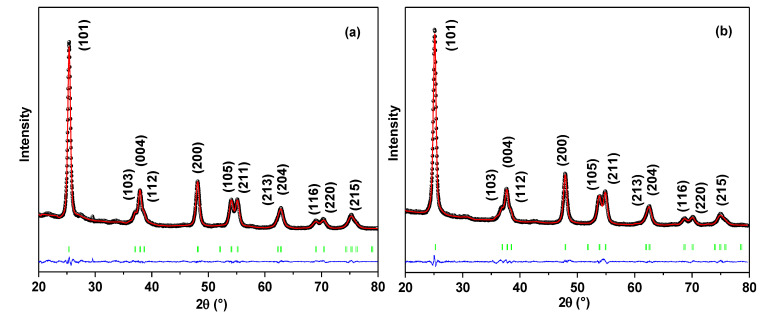
Rietveld refinement of X-ray diffractograms of TiO_2_ anatase after adsorption of Pb(II) for C_o_ = 47.54 mg L^−1^ (**a**) and Pb C_o_ = 1.16 mg L^−1^ (**b**). The black dots (I_obs_) represent the experimental data, the red lines (I_cal_) are the calculated XRD diffractogram, the green vertical lines are the Bragg positions, and the blue lines represent the residual lines.

Figure 1a,b shows the refined X-ray diffractograms of nano-TiO_2_ with the corresponding refinement model after the Pb adsorption process with initial concentrations of 47.54 mg L^−1^ (TiO_2_-PbC1) and 1.16 mg L^−1^ (TiO_2_-PbC2). Only the pure anatase phase was observed; the refined parameters are summarized in Table 1. Considering anisotropic size broadening, the mean apparent crystallite sizes were calculated using Scherrer’s formula, which may be expressed as a linear combination of spherical harmonics and is provided in the FullProf Suite program [30]. The data corroborated the presence of the tetragonal crystal structure of the anatase phase even after adsorption of Pb(II) for both initial concentrations. The average apparent size of anatase crystallites was 9.9 nm and 8.7 nm, evidencing that there was not much variation in the microstructural parameters (Table 1), even with increasing concentration of Pb. Anatase crystallite size values of 10.1 nm have been reported by Canchanya et al. and of 12.22 nm by Patidar and Jain, with no application in the adsorption process. In comparison with these values, the crystallite size of anatase showed a slight decrease after the Pb(II) adsorption process [31,32].

#### 2.1.2. SEM and EDS Mapping Analysis

**Figure 2:**
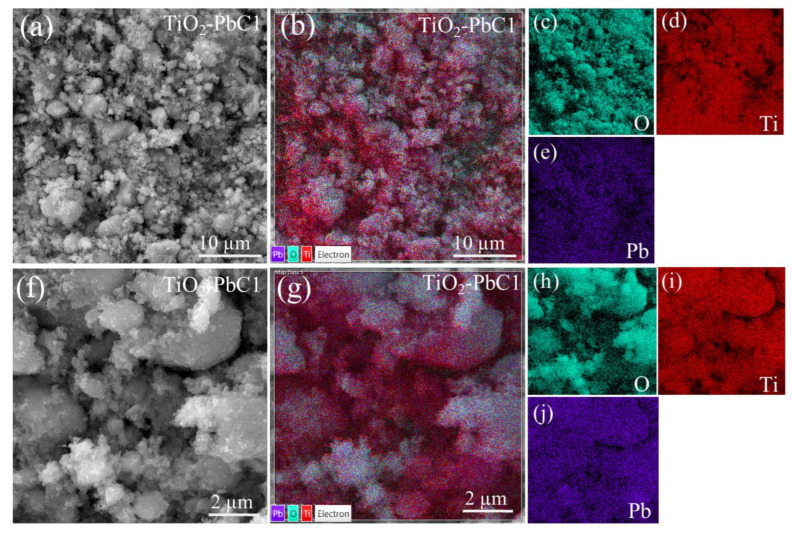
(**a**,**f**) SEM images, (**b**,**g**) EDS mapping images, and (**c**–**e**,**h**–**j**) elemental EDS images for nano-TiO_2_ with C_0_ (Pb(II)) = 47.54 mg L^−1^.

Figure 2 and Figure S1 depict the SEM images for two magnifications (a and f), where characteristic granular morphologies of nano-TiO_2_ powders exposed to two different Pb(II) concentrations are observed. EDS mapping images are observed in Figure 2 and Figure S1b,g and the elemental images for O (c,h), Ti (d,i), and Pb (e,j) are shown. The presence of Pb(II) was corroborated at low and high initial concentrations. Table 2 summarizes the weight percentage for the identified elements after analyzing the EDS spectrum (see Figure S2).

#### 2.1.3. TEM Analysis

**Figure 3:**
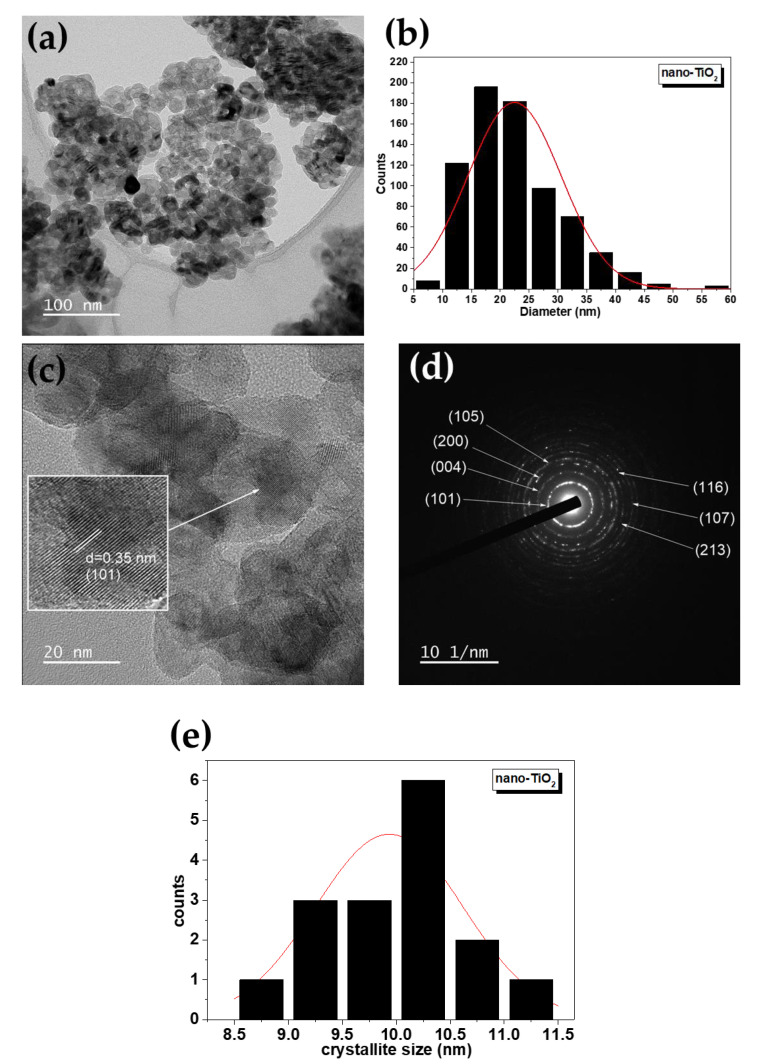
(**a**) TEM image of TiO_2_-PbC1 NPs (bar length of 100 nm), (**b**) PSD histogram for nano-TiO_2_-PbC1, (**c**) Zoomed TEM image of nano-TiO_2_-PbC1, (**d**) SAED pattern of nano-TiO_2_-PbC1, and (**e**) crystallite size distribution histogram for nano-TiO_2_-PbC1 obtained from Rietveld refinement. C_0_ (Pb(II)) = 47.54 mg L^−1^.

Figure 3 and Figure S3 show the effect of adsorption of Pb(II) on nano-TiO_2_ morphology at different initial adsorbate concentrations of 47.54 mg L^−1^ and 1.16 mg L^−1^, with the aim to study the morphology and particle size distribution (PSD). Figure 3a and Figure S3a show that the nano-TiO_2_ particles presented mostly spherical morphology [33]. In addition, the PSD was plotted from the count of 700 NPs, from which average particle sizes of 22.46 nm (Figure 3b) and 17.1 nm (Figure S3b) were estimated. The adsorption of Pb(II) with a higher initial concentration showed a slight increase in particle size compared to the TiO_2_ NP size, without application in the adsorption process, of 17.9 nm reported by Canchanya et al. [31]. With a low initial concentration there was no variation in nano-TiO_2_ particle size. The PSD histogram makes it obvious that the NPs had a wide PSD and that their distribution closely followed a normal curve. The selected area electron diffraction (SAED) patterns are presented in Figure 3d and Figure S3d. The crystalline planes of nano-TiO_2_ are specified by the Miller indices, which were identified from the pattern rings, which agree with the XRD results (Figure 1). Looking at the images, it can be suggested that the Pb adsorbed on nano-TiO_2_ formed a polynanocrystalline structure [33,34]. Figure 3c and Figure S3c show the crystal lattice planes of nano-TiO_2_, for both samples; a crystal lattice plane (110) with a d-spacing of 0.35 nm was estimated. On the other hand, the crystallite domain size distribution gave values of 9.9 nm (Figure 3e) and 8.7 nm (Figure S3e).

#### 2.1.4. XPS Analysis

**Figure 4:**
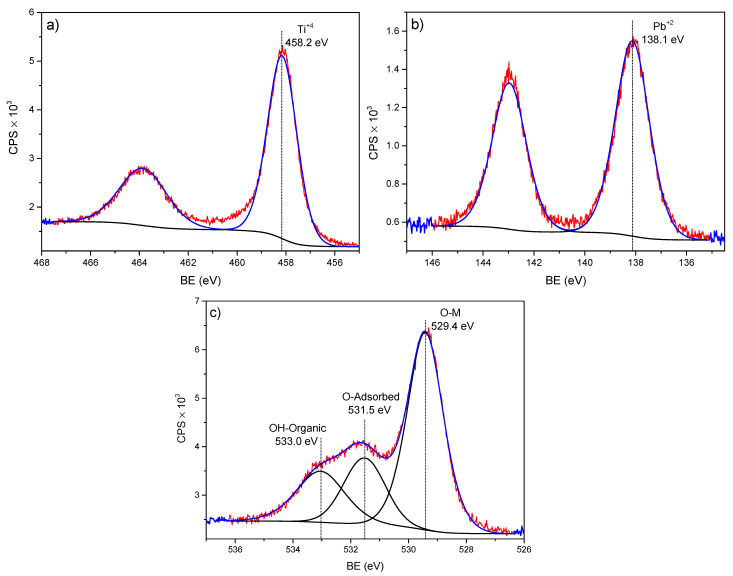
XPS spectra of the TiO_2_-PbC1 sample, showing the deconvoluted peaks of: (**a**) Ti 2p, (**b**) Pb 4f, and (**c**) O 1s.

**Figure 5:**
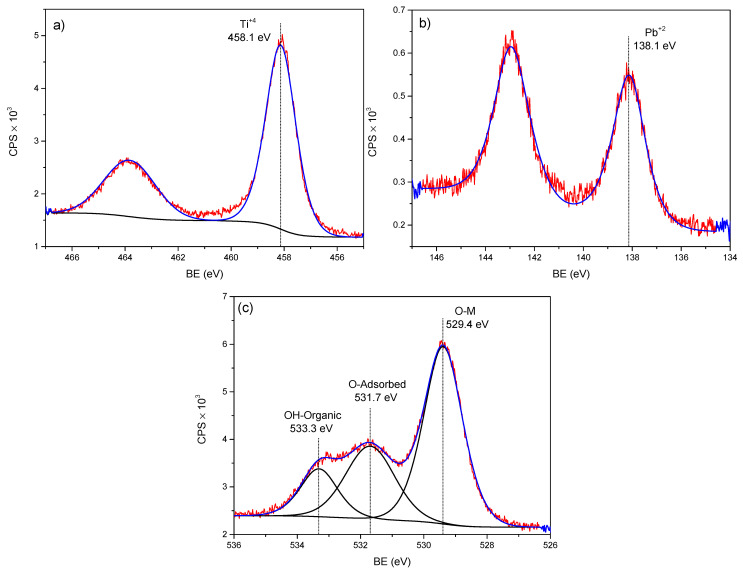
XPS spectra of the TiO_2_Pb-C2 sample, showing the deconvoluted peaks of: (**a**) Ti 2p, (**b**) Pb 4f, and (**c**) O 1s.

Figure 4 and Figure 5 show the XPS spectra for low- and high-concentrated Pb adsorbed onto the nano-TiO_2_ sample—in principle, there was no alteration in the BE of Ti 2p_3/2_ with respect to both Pb concentrations. Furthermore, by increasing the concentration of the metal (Pb) of the aqueous solution (from 1.16 mg L^−1^ to 47.54 mg L^−1^) in the nano-TiO_2_, no increases in at%. Pb adsorbed were observed (Table 3). This indicates a saturation limit of adsorption of Pb atoms by the TiO_2_ NPs. Regarding the energy analysis of Ti 2p_3/2_, this indicated that TiO_2_ with a rutile or anatase structure (indistinguishable by XPS) [35] went through an oxidation process with respect to its initial structure. This was due to the adsorption process and was confirmed by the BE of O 1s at 531.7 eV, as suggested by Lu et al. [36].

### 2.2. Adsorption Kinetics

In order to find the time in which maximum removal is obtained, the adsorption kinetic performance was evaluated at pH 5.5, 298 K and a dose of 1 g L^−1^. This removal percentage was calculated by applying the following Equation (1):

(1) $$R\left(\%\right)=\frac{C_{0}-C_{f}}{C_{0}}\times 100\%$$

**Figure 6:**
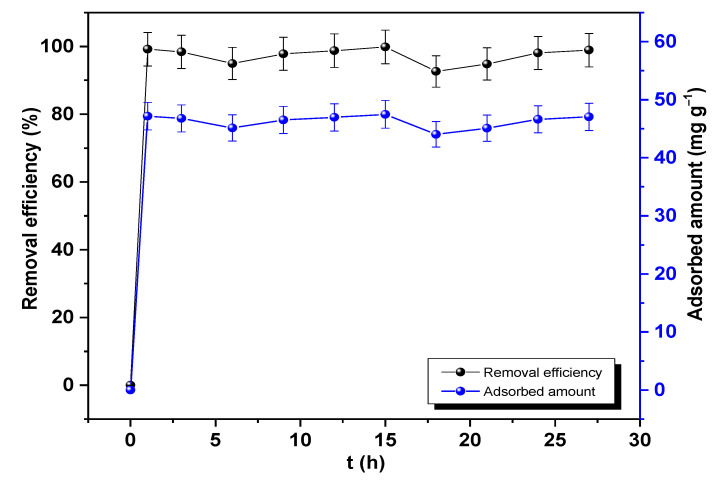
Evaluation of time on the efficiency and adsorbed amount of Pb(II) onto nano-TiO_2_.

Figure 6 shows that the percentage removal of Pb(II) on nano-TiO_2_ was higher than 92% in all cases; with longer time, there were only slight variations. Therefore, the equilibrium time was set at 1 h with 99.18% Pb removal. This time frame was shorter than the value found by Poursani et al., who determined the equilibrium time to be 4 h at an adsorbent dose of 3 g L^−1^ [39]. Moreover, the study of Recillas et al. reported an equilibrium time of 0.62 h at 1 g L**^−^**^1^ [40]. Jyothi et al. achieved 70% Pb removal with a dose of 0.4 g L**^−^**^1^ [6]. Finally, Inquil and Valverde achieved an efficiency of 79.58% in river water with a concentration of Pb 2.36 mg L**^−^**^1^, 0.6 g L**^−^**^1^ of nano-TiO_2_, and 1 h of interaction [41]. These results indicate that the equilibrium time estimated in this work is smaller than that reported by other authors (Table 4).

Therefore, it was shown that nano-TiO_2_ has high potential for Pb(II) adsorption by reducing the concentration from 47.54 mg L**^−^**^1^ to 0.39 mg L**^−^**^1^. Regarding the adsorbed amount, it was also constant from 1 h of interaction time, with the maximum adsorbed amount of 47.47 mg g**^−^**^1^ at 15 h (see Figure 6).

The maximum adsorption capacity determined in this work was 65.99 mg g^−1^. This result is lower than the 194.28 mg g**^−^**^1^ obtained by Kanna et al., where an initial concentration of Pb(II) of 400 mg L**^−^**^1^, pH 7, and an adsorbent dose of 0.50 g L**^−^**^1^, were used [42].

**Table 4:** Maximum capacities and adsorption percentages of different adsorbents towards Pb(II) in aqueous solutions.

Adsorbent	Adsorption Capacity q_m_ (mg g^−1^)	pH	Time (h)	Dose (g L^−1^)	C_i_ (mg L^−1^)	Reference
nano-TiO_2_	32.03	8	2	0.01	0.1	[43]
nano-TiO_2_	7.41	6	4	4	25	[39]
nano-TiO_2_	158.73	7	24	0.32	100	[40]
Anatase	194.28	7	0.25	$2\times 10^{-3}$	400	[42]
Titania nanofiber	2.56	5	4	0.05	0.50	[44]
Composed of pectin and nano-TiO_2_	66.28	5.5	1	3	95.83	[4]
Graphene oxide and TiO_2_ nanocomposite	65.6	5.6	12	0.025	50	[45]
Graphene oxide nanocomposite	35.6	5.6	6	0.025	50	[45]
nano-ZnO	6.7	6.6	72	1	1010	[46]
nano-CeO_2_	9.2	7	NR	2	NR	[47]
Modified nano-Al_2_O_3_	100	5	1.5	1	50	[48]
Kaolinite	7.75	4.5	48	0.1	2000	[49]
Montmorillonite	31.10	5.7	3	2	50	[50]
Bentonite	51.19	NR	3	20	200	[51]
Biomass of *A. bisporus*	33.78	5.0	4	3	100	[52]
Biomass of *Aspergillus niger*	32.60	4.0	2	1	100	[53]
Anaerobic granular biomass	255.00	4.0–5.5	0.5	10	100	[54]
nano-TiO_2_	65.99	6.5	1	1	47.54	This work.

NR: Indicates not reported.

In Table 4, a simple comparison of some adsorbents and nanoadsorbents for metal removal has been made. The adsorption capacity obtained in this work was higher than that of biomass-based adsorbents, except for anaerobic granular biomass [54]. Likewise, nanoadsorbents that have adsorption capacity values above nano-TiO_2_ are modified nano-Al_2_O_3_ and pectin-TiO_2_ nanocomposite. On the other hand, regarding the works carried out with nano-TiO_2_, Recillas et al. obtained an adsorption capacity of 158.73 mg g**^−^**^1^, which may be higher due to the contact time and initial concentration conditions, which were 24 h and 100 mg L**^−^**^1^, respectively [40]. Subsequently, Kanna et al. found an adsorption capacity of 194.28 mg g**^−^**^1^ using anatase nanoparticles (neutral pH), a concentration of 400 mg L**^−^**^1^, and a minimal dose of 2 × 10**^−^**^3^ g L^−1^. Remarkably, our work employed a similar minimum dose of adsorbent and achieved comparable results, demonstrating the effectiveness of this low-dose approach [42]. It is worth noting that the advantages of TiO_2_ NPs lie mainly in their high removal efficiency reported in previous studies, high specific surface area, low cost, availability, low environmental impact, and chemical activity, which means that modification of their adsorption capacity has been widely studied [5,19,55]. On the other hand, they should be considered as an eco-sustainable material because they can be reused by means of desorption. Evidence provided by Hu and Shipley indicates optimal desorption at pH 2 and up to eight desorption cycles; furthermore, an EDTA solution could be used for this purpose [56].

The kinetic behavior of Pb adsorption was investigated by analyzing the adsorption rate using linear and nonlinear PFO, PSO, E, and IDM, which are based on the adsorption equilibrium capacity (see Table S1 and Table 5). In this work, linear and nonlinear models were compared since it is known that linear models can generate errors as a consequence of transformations from nonlinear to linear forms, implying that they can be inaccurate. Therefore, we prefer to compare with the nonlinear forms to produce accurate parameter results [57].

Four different kinetic adsorption models were applied to examine the kinetic behavior of Pb(II) adsorption (Figure S4). These models are based on equilibrium adsorption capacity, which is used to analyze the adsorption rate. The PFO equation’s constant, k_1,_ yielded a value of 0.001 h^−1^; however, the intercept was q_e_ = 0.69 mg g^−1^, which was not the same as the value for q_e_ found in the kinetic adsorption tests (q_e_ = 47.15 mg g^−1^). Additionally, the R^2^ was close to 0, which further supports the lack of linearity. Therefore, it is concluded that the PFO, IDM, and E kinetic models cannot reproduce the results of kinetic adsorption. Instead, by applying the PSO model, the values k_2_ = 0.58 g mg^−1^ h^−1^ and q_e_ = 46.29 mg g^−1^ were obtained, which correspond to the equilibrium adsorption capacity determined experimentally. The obtained R^2^ value of 0.99 affirms the nearly perfect linearity depicted in Figure S4b. This result suggests that the Pb(II) adsorption kinetics mechanism is governed by the PSO equation.

**Figure 7:**
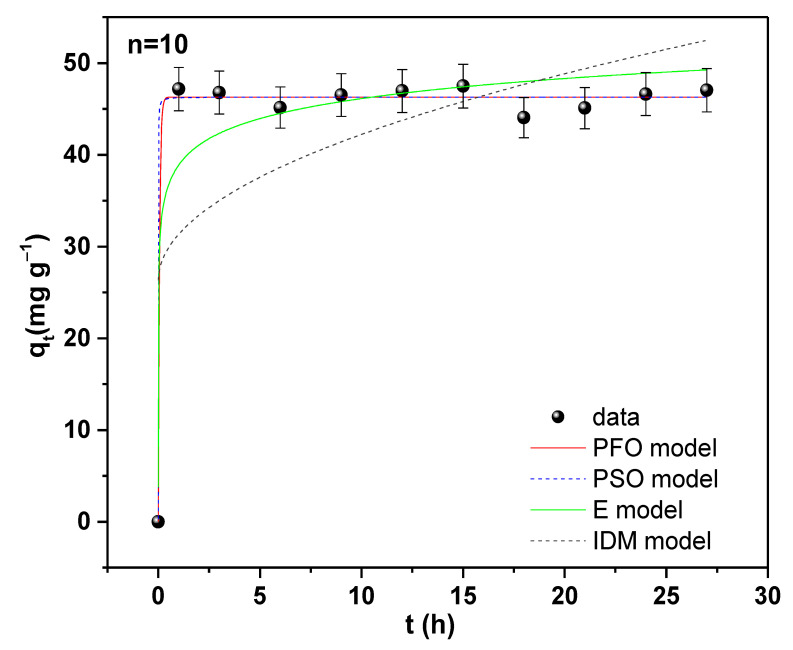
Nonlinear models of Pb(II) adsorption kinetics on nano-TiO_2_. Adsorbent dose of 1 g L^−1^, pH 5.5, C_o_ = 47.54 mg L^−1^.

The PSO lineal fit model had a capacity of 46.29 mg g^−1^, equal to the value obtained by the nonlinear fit model. However, the velocity constant (k_2_) for the linear model was very low at 0.58 g mg^−1^ h^−1^ compared to the nonlinear fit of 12.74 g mg^−1^ h^−1^. Consequently, the linear model did not fit the experimental data well. On the other hand, the nonlinear models of PFO, PSO, and E presented a good fit to the experimental data with R^2^ greater than 0.92, except for the IDM model (see Figure 7). Therefore, in order to find the best fit, the BIC was calculated with Equation (S12) in the Supplementary Materials [58]. The PFO and PSO models yielded the lowest BIC values, with a difference between the two values of less than 2 (Table 5), so both models can represent the adsorption process of Pb on TiO_2_. In this regard, numerous authors have suggested that the PSO model best describes the adsorption procedure [39,59,60]; hence, the chemisorption process is the predominant one in the adsorption process.

### 2.3. Effect of pH

**Figure 8:**
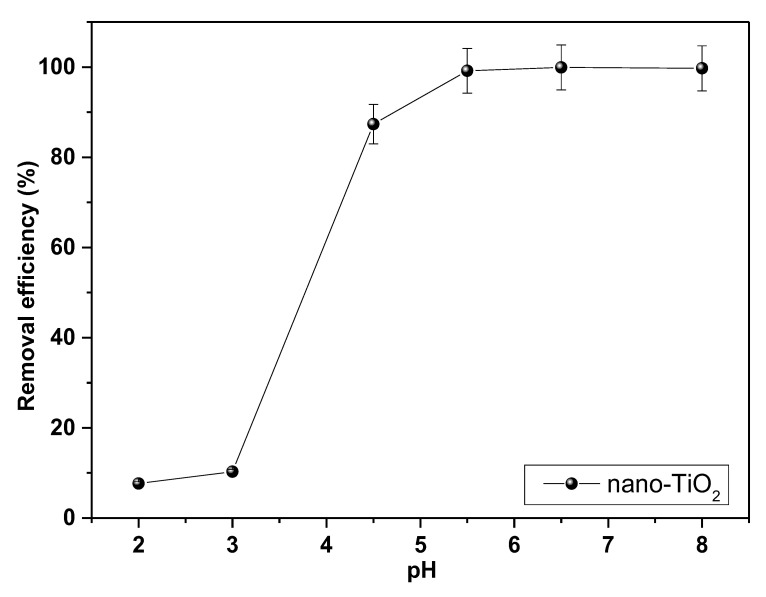
pH dependence of the Pb removal on nano-TiO_2_.

The pH is one of the most important adsorption parameters since it alters the charge on the adsorbent surface, causing a significant change in its adsorption capacity, in addition to affecting the existing form of Pb in the solution [61]. Figure 8 shows a low percentage of adsorption at low pH values of 2 to 3. In this range, the H^+^ ions are found with a higher concentration in the solution and mobility on the surface, generating competition with the Pb^2+^ ions for the adsorption sites, generating a decrease in the adsorption percentage [61]. The gradual increase in pH causes a decrease in H^+^ ions and an increase in hydroxyl groups on the nano-TiO_2_ surface due to the protonation-dissociation equilibrium [62]. This increases the active adsorption sites available for interaction with Pb^2+^ ions and promotes the electrostatic attraction and ion exchange that occurs between the positive charges of the metal ions and the negative charges on the adsorbent surface [63]; these lead ions can form species such as Pb(OH)^+^, Pb_2_(OH)^3+^, Pb_3_(OH)^4+^, and others [64].

Increasing the pH generated an increase in the adsorption efficiency, reaching a maximum percentage of adsorption at 6.5 with a value of 99.93%. In the same way, other studies obtained similar results for pH in the elimination of Pb with nano-TiO_2_ [27,39,40,56]. At pH values above the point of zero charge (pH_PZC_), for nano-TiO_2_ located at 6.5 [65], there was no significant reduction in the adsorption percentage since, with increasing pH, the nano-TiO_2_ surface increased its negative charge favoring adsorption [60]. However, the experiments were not conducted at pH levels greater than 8 due to the progressive precipitation of Pb and formation of complexes that prevented its adsorption [62].

### 2.4. Adsorbent Dose

**Figure 9:**
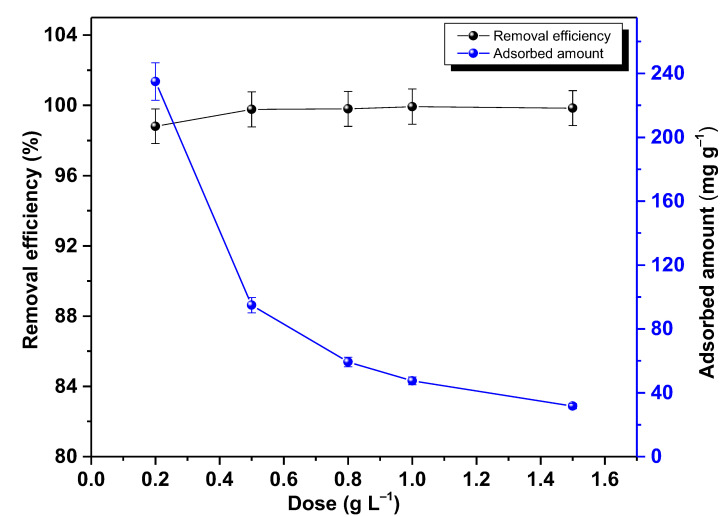
Adsorbent dose dependence of the Pb removal and adsorbed amount for nano-TiO_2,_ equilibrium time of 1 h.

Figure 9 illustrates the relationship between the Pb(II) removal efficiency and adsorbed amount against nano-TiO_2_ adsorbent dose. It was observed that the percentage of adsorption increased as the absorbent dose increased, given that, with a greater amount of adsorbent, the number of adsorption active sites on the nano-TiO_2_ surface increases and the competition between ions occupying the active sites reduces, as a result there is an increase in the adsorption of Pb ions [60,62]. On the other hand, the adsorbed amount decreases with increase in the dose since the amount of adsorbent is increased but the amount of solute in the solution remains constant [66]. This prevents reaching the maximum amount of adsorption in an active site. When the dose of the adsorbent coincides with the total amount of Pb^2+^ ions in the solution, the adsorbed amount reaches the highest possible value [59], in this case, 99.93% of Pb was removed from the water with a dose of 1 g L^−1^; Giammar et al. reported similar results [27]. After that, by further increasing the adsorption dose, the removal percentage decreased slightly.

### 2.5. Adsorption Isotherms

**Figure 10:**
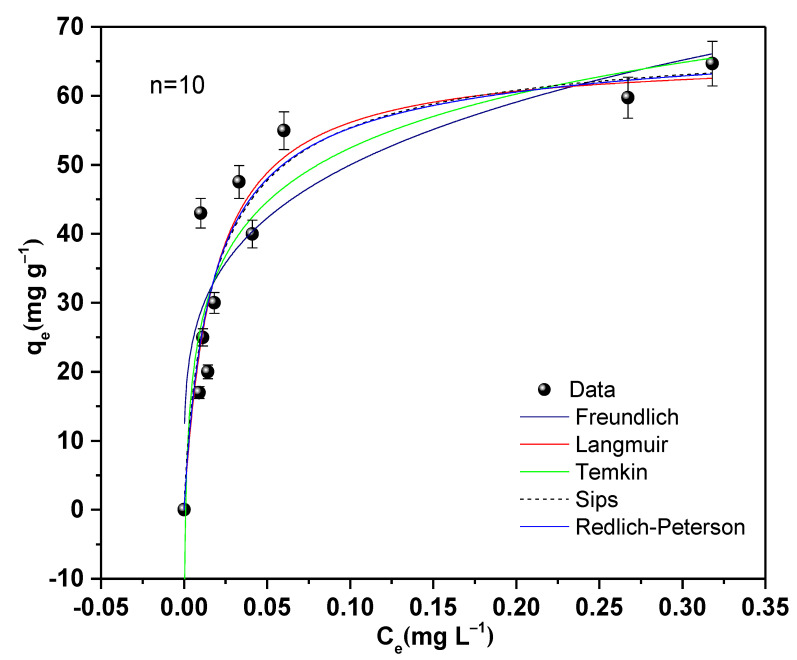
Pb(II) adsorption isotherms from irrigation water using nano-TiO_2_.

The adsorption isotherms obtained for the TiO_2_ NPs are plotted in Figure 10. An increase in adsorption capacity can be seen with increasing initial adsorbate concentration. The optimal conditions for obtaining the adsorption experimental data were a pH of 6.5, a dose of 1 g L^−1^, and 1 h of agitation at equilibrium. The adsorption parameters calculated from the fit, and the BIC and RSS values are presented in Table 6. Evidently, the Freundlich model yielded the lowest value of R^2^, so it was ruled out, implying that the process is not governed by multilayer. The other models had high values close to each other of R^2^. Therefore, another criterion by which to evaluate the models is the BIC value, where the lowest values were found for the Langmuir and Sips models; the difference between the BIC values of the two models was less than 2. In fact, both applied models could explain well the adsorption process of Pb(II) on nano-TiO_2_ [67]. The Langmuir model implies that the process is characterized by adsorption on the homogeneous surface sites of the adsorbent by the formation of a monolayer of adsorbate, with all sites being identical and having equivalent energies [39]. Likewise, the Sips model supports the Langmuir model—this was inferred from the value of m_s_ = 0.85, close to unity, which confirms homogeneous adsorption [68]. It was also verified by the value of β = 0.97, close to 1, obtained by the Redlich–Peterson model, which indicates a monolayer reaction [69]. The maximum adsorption capacity predicted by the Langmuir model was 65.99 mg g^−1^, close to the value computed by the Sips model of 69.22 mg g^−1^. After comparing the adsorption capacity of TiO_2_, it was confirmed that it was superior to other adsorbents reported in the literature (Table 4).

### 2.6. Adsorption Mechanisms of Pb(II) on TiO_2_

High Pb adsorption was seen at values of pH above pH_PZC_ = 6.5, because the adsorbent surface was negatively charged, which generated an increase in the Coulomb interaction between the ions and the adsorbent surface. In their study, Chen et al. showed how H_2_O adsorbs to TiO_2_ by bond breaking and formation, which is referred to as chemisorption. Their system demonstrated how the (O-H) bond in water dissociated and created new bonds that connected the H/O atom of H_2_O and the O/Ti atom of TiO_2_, which included forming the O-H bond on the surface of TiO_2_ [70]. Therefore, the ion exchange mechanism described by Equations (2) and (3) can be used to explain the adsorption of Pb(II) on nano-TiO_2_ as the binding of Pb^2+^, Pb(OH)^+^, and Pb(OH)_2_ with the active sites available on the surface of the adsorbents.

(2) $$\begin{array}{cc} 2\left(-ROH\right)+{Pb}^{2+}\to 2\left(RO\right)Pb+2H^{+} & \text{Step 1} \end{array}$$

(3) $$\begin{array}{cc} -ROH+{Pb\left(OH\right)}^{+}\to \left(-RO\right)Pb\left(OH\right)+H^{+} & \text{Step 2} \end{array}$$

or by hydrogen bonding given by Equation (4)

(4) $$\begin{array}{cc} 2\left(-ROH\right)+Pb{\left(OH\right)}_{2}\to {\left(-ROH\right)}_{2}+Pb{\left(OH\right)}_{2} & \text{Step 3} \end{array}$$

**Figure 11:**
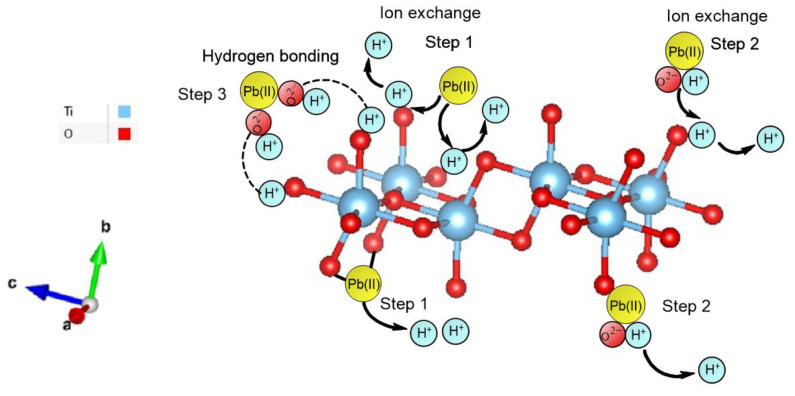
Pb(II) adsorption mechanisms on nano-TiO_2_.

Since the oxygen of the (O-H) on the adsorbent’s surface is a strong Lewis base by its characteristic vacant doublet electrons, the oxygen forms a coordination complex with the low electron chemical species, such as metal ions [60,71], as shown in Figure 11. Step 1 and step 2 reveal the ion exchange first stage (deprotonation) and the adsorption of Pb^2+^ and Pb(OH)^+^ on the deprotonated surface active sites, respectively, expressed in Equations (2) and (3). Step 3 is the formation of hydrogen bonds, expressed in Equation (4).

## 3. Perspectives

For further studies, we intend to continue with the application of nano-TiO_2_ in water bodies with natural organic matter (NOM) since it has been found that nano-TiO_2_ with ions such as Pb^2+^ and Ca^2+^ adsorbed provides more complexation sites, which may favor the adsorption of NOM through the formation of bridges between the nano-TiO_2_ and NOM surface [72,73].

On the other hand, in the search to optimize the equilibrium time and the acceleration of the adsorption kinetics, it is intended to modify the surface properties of nano-TiO_2_ by adding materials such as graphene oxide [45], pectin [4], chitosan [74], carbon nanotubes, montmorillonite, binary oxides, and ternary compounds with magnetite and silica [75], whose adsorbents have a higher adsorption capacity, reaching equilibrium times of less than 1 h.

Considering that, after the adsorption process, the nano-TiO_2_ with Pb(II) ion species on its surface cannot be released into the environment due to its toxicity, the reusability of the nano-TiO_2_ and the recovery of the adsorbed metal are important, making the adsorption process more cost-effective and environmentally friendly. Hu and Shipley [56] investigated the regeneration capacity of nano-TiO_2_ for Pb(II) recovery using various salts, 95% of desorbed Pb(II) being obtained by use of NaNO_3_. On the other hand, EDTA was used for optimal regeneration of nano-TiO_2_, which can be reused even for removal in systems with various metals [56,76]. According to Engates and Shipley [43], nano-TiO_2_ could be reused in eight cycles of Pb(II) adsorption, in which a constant and similar adsorption capacity was achieved in each cycle. With respect to the use of desorbed Pb(II) as Pb(OH)_2_, this has applications in the field of chemical catalysis [77].

## 4. Materials and Methods

### 4.1. Materials and Chemicals

All the chemical reagents utilized were of analytical quality. TiO_2_ NPs were acquired from Sigma Aldrich (Darmstadt, Germany) and used unpurified. SCP Science (Quebec, QC, Canada) provided a Pb(II) standard solution with a concentration of 999 ± 3 µg mL^−1^. Merck (Darmstadt, Germany) brand hydrochloric acid (HCl) and sodium hydroxide (NaOH) reagents were used.

Water polluted with Pb was collected from the irrigation canal on the left bank of the Mantaro river (CIMIRM) at El Mantaro, Jauja, Peru, at 3320 m above sea level and 11°41′21.5″S 75°22′24.5″W. The original Pb(II) concentration of 0.0178 mg L^−1^ in the water was modified to 47.54 mg L^−1^. In addition, other heavy metals were found in the water, whose concentrations were compared to the maximum concentration established (presented in parentheses) by the Food and Agriculture Organization (FAO) for irrigation water [78]: As: 0.0176 mg L^−1^ (0.1 mg L^−1^), Cu: 0.021 mg L^−1^ (0.2 mg L^−1^), Ni: 0.0032 mg L^−1^ (0.2 mg L^−1^), Cd: 0.00047 mg L^−1^ (0.01 mg L^−1^), Cr: 0.0026 mg L^−1^ (0.1 mg L^−1^), Zn: 0.1305 mg L^−1^ (2 mg L^−1^) and Fe: 2.8477 mg L^−1^ (5 mg L^−1^). As can be seen, there were no concentrations above the limits established by FAO.

### 4.2. Characterization of Nano-TiO_2_

#### 4.2.1. X-ray Diffraction (XRD)

XRD was used to characterize the materials using an Empyrean diffractometer with CuK_α_ radiation at wavelength = 1.5406 Å. The X-ray diffractograms were taken following a step scanning arrangement with a range of 2θ = 20–80° and a resolution of 0.01° per step. Match V3 software was used to identify the crystallographic phase of anatase nano-TiO_2_, with the tetragonal crystalline structure, space group I41/amd, and cell parameters a = 3.7892 Å and c = 9.5370 Å as an initial matching candidate, with the crystallographic information files (CIF) #5000223 for the anatase phase. The peak’s profile function Thompson–Cox–Hastings pseudo-Voigt axial divergence asymmetry was used for the Rietveld refinement using the program FullProf Suite (version January 2021). Furthermore, the aluminum oxide Al_2_O_3_ standard was used to determine the diffractometer’s instrumental resolution function (IRF), which yielded Caglioti coefficients including U = 0.0093, V = 0.0051, and W = 0.0013 [31].

#### 4.2.2. Scanning Electron Microscopy (SEM), Energy Dispersive Spectroscopy (EDS), and Transmission Electron Microscopy (TEM)

SEM and TEM techniques were employed to estimate the mean size of particles and the TiO_2_ morphologies. To assess the atomic composition, the elemental compositions of the materials were examined using EDS mapping.

#### 4.2.3. X-ray Photoelectron Spectroscopy (XPS)

Chemical surface examinations on the studied materials were carried out utilizing the SPECS PHOIBOS 100/150 equipment with a hemispheric analyzer spectrometer, operating at 1486.6 eV of Al Kα. The XPS spectra were obtained using a high-resolution polychromatic X-ray source with a 0.02 eV energy step. The SPECS company’s Casa-XPS software was used to change the peak locations of the Ti 2p, O 1s, and Pb 4f levels to compute the chemical binding energies (BE) of produced species, as well as the relative atomic amounts on the sample surfaces. The spectra were calibrated using adventitious carbon (B.E. reference) at C 1 s = 284.6 eV after being calibrated with an electron flood cannon at 12 µA and 1 eV.

### 4.3. Pb(II) Removal Experiments with Nano-TiO_2_

The Pb(II) removal experiments were carried out in real water samples contaminated with Pb, taken from the CIMIRM-Junín-Perú irrigation canal, whose initial concentration was modified from 0.0178 mg L^−1^ to 47.54 mg L^−1^. To modify the concentration of this metal, a dilution was carried out from a standard solution of Pb at an initial concentration of 999 ± 3 µg mL^−1^. Pb quantification, in all cases, was performed using the inductively coupled plasma mass spectrometry (ICP-MS) analysis technique.

#### 4.3.1. Adsorption Kinetics

Pb(II) adsorption kinetics experiments with nano-TiO_2_ were performed in order to determine the equilibrium time and evaluate the kinetic models.

Ten experiments were carried out depending on the contact time, in a range from 1 to 27 h, at 47.54 mg L^−1^ of initial concentration of Pb(II), using a nano-TiO_2_ dose of 1 g L^−1^, at pH 5.5, under constant stirring at 300 rpm, at room temperature.

#### 4.3.2. pH Effect

In order to know the effect of pH on the removal of Pb(II) using nano-TiO_2_, experiments were carried out controlling pH in the range of 2 to 8, for a stirring time of 1 h (equilibrium time determined by the experiments of adsorption kinetics), with an adsorbent dose of 1 g L^−1^, maintaining constant stirring at 300 rpm at room temperature. The control of the previously indicated pH range was performed by adding drops of HCl and NaOH solutions in concentrations of 0.05 M, 0.1 M, 1 M, and 4 M.

#### 4.3.3. Adsorbent Dose

The adsorbent dose experiments were carried out on real water samples at an initial Pb(II) concentration of 47.54 mg L^−1^. The nano-TiO_2_ dose range considered in this work was from 0.2 g L^−1^ to 1.5 g L^−1^. The other experimental parameters were: pH 6.5, constant stirring time of 1 h at 300 rpm at room temperature.

#### 4.3.4. Adsorption Isotherms

Pb(II) adsorption isotherms with nano-TiO_2_ were constructed using the previously determined experimental conditions: the equilibrium time, the optimum pH, and the adsorbent dose. Utilizing these values, ten tests were conducted on irrigation canal water samples with beginning concentrations ranging from 3 to 65 mg L^−1^. To analyze the data obtained, the Langmuir, Freundlich, Redlich–Peterson, Sips and Temkin adsorption models were used.

The theoretical description of the kinetic and isotherm models is given in the Supplementary Materials.

## 5. Conclusions

The removal efficiency of Pb(II) using nano-TiO_2_ in irrigation water was evaluated at a modified initial concentration of 47.54 mg L^−1^, in the presence of other metals. This nanomaterial was characterized by XRD after adsorption, confirming the presence of the tetragonal crystalline structure of the anatase phase. The mean crystallite size was obtained by Rietveld refinement giving a value of 9.9 nm; the mean particle size was determined by TEM, being 22.46 nm. The XPS studies indicated that there was no alteration of the absorption bands with respect to increase in the concentration of Pb(II). The SEM images showed changes in the surface morphology of the nano-TiO_2_, confirming the occurrence of metal adsorption. In the Pb(II) removal experiments, more than 99% Pb(II) elimination was achieved by determining the optimal contact time, pH, and dose of adsorbent, having values of 1 h, 6.5, and 1 g L^−1^, suggesting that there was no significant influence of the prevalence of other metal ions. The experimental data were evaluated with linear and nonlinear models of adsorption kinetics. The best fit was found to correspond to the PSO model, indicating that the process was governed by chemisorption. This result was supported by evaluation of the adsorption isotherms, where the Langmuir and Sips models provided the best fit, evidencing that adsorption occurred in the homogeneous sites of the nano-TiO_2_ surface through the formation of a adsorbate monolayer. In addition, a high adsorption capacity of nano-TiO_2_ for Pb(II) of 65.99 mg g^−1^ was estimated, higher than the values reported in other studies. In general, the results of this study indicate that nano-TiO_2_ is a potential adsorbent for application in the treatment of water contaminated with Pb(II).

## Figures and Tables

**Table 1:** Rietveld refinement parameters of the TiO_2_-PbC1 and TiO_2_-PbC2 samples: lattice parameters, cell volume, and Caglioti values. Agreement R-factors R_exp_ (%), R_p_ (%), R_wp_ (%), and goodness of fit, chi-square (χ^2^).

Refined Parameters	TiO_2_-PbC1 C_o_ = 47.54 mg L^−1^	TiO_2_-PbC2 C_o_ = 1.16 mg L^−1^
a(Å)	3.782 (9)	3.798 (9)
b(Å)	3.782 (9)	3.798 (9)
c(Å)	9.488 (3)	9.527 (3)
α(°)	90	90
β(°)	90	90
γ(°)	90	90
$V({Å}^{3})$	135.750 (6)	137.441 (6)
$Y_{00}$	−0.077 (7)	0.022 (4)
$Y_{20}$	0.675 (8)	0.972 (8)
$Y_{40}$	0.650 (9)	0.180 (9)
$Y_{44+}$	0.045 (6)	−0.127 (5)
$Y_{44-}$	−0.958 (2)	−0.585 (2)
$Y_{60}$	−0.120 (9)	0.554 (9)
$Y_{64+}$	−0.368 (4)	−0.184 (3)
$Y_{64-}$	−2.311 (2)	0.952 (2)
FWHM parameters		
U	4.464	4.342
V	−2.569	−2.549
W	0.907	0.919
Average max strain	108.48 (4)	98.63 (3)
Average size (nm)	9.9 (7)	8.7 (5)
$R_{exp}(\%)$	4.52	4.54
$R_{p}(\%)$	7.21	6.90
$R_{wp}(\%)$	7.70	7.38
χ2	2.90	2.63

**Table 2:** Elemental composition for the studied samples obtained from EDS spectrum analysis.

Sample	Ti (% wt)	O (% wt)	Pb (% wt)
TiO_2_-PbC1	47.7 ± 0.2	47.5 ± 0.2	4.8 ± 0.1
TiO_2_-PbC2	57.8 ± 0.2	37.3 ± 0.2	4.8 ± 0.1

**Table 3:** Result of the fit for the XPS peaks of the Pb(II) adsorbed nano-TiO_2_ samples.

Sample	Level	BE (eV)	at.%	Bond Type	Ref.
	O 1s	529.4	45.2	O-Metal	[37]
	O 1s	531.7	22.7	O-adsorbed	[36]
TiO_2_-Pb C2	O 1s	533.3	11.6	OH-Organic	[38]
1.16 mg L^−1^	Ti 2p_3/2_	458.1	18.4	Ti^+4^ (TiOx)	[35,36]
	Pb 4f_7/2_	138.1	2.1	Pb^+2^	[36]
	O 1s	529.4	46.6	O-Metal	[37]
TiO_2_-Pb C1	O 1s	531.5	17.4	O-adsorbed	[36]
47.54 mg L^−1^	O 1s	533.0	16.2	OH-Organic	[38]
	Ti 2p_3/2_	458.2	17.7	Ti^+4^ (TiOx)	[35,36]
	Pb 4f_7/2_	138.1	2.1	Pb^+2^	[36]

**Table 5:** Fit parameters of nonlinear models of adsorption kinetics of PFO, PSO, E, and IDM.

PFO Model		PSO Model		E Model	
q_e_ exp (mg g^−1^)	47.15	q_e_ (mg g^−1^)	46.29 (4)	β (g mg^−1^)	0.32 (4)
q_e_ (mg g^−1^)	46.28 (4)	k_2_ (g mg^−1^ h^−1^)	12.74 (5)	α (mg h^−1^)	43,223,954.34 (1)
k_1_ (h^−1^)	16.29	h (mg g^−1^ h^−1^)	27,298.58		
R^2^	0.99	0.99		0.92	
RSS	11.38	11.38		126.33	
BIC	5.90	6.02		29.96	
**IDM**					
k_p_ (mg g^−1^ h^−0.5^)	5.04 (2)				
C_1_ (mg g^−1^)	26.28 (8)				
R^2^	0.29				
RSS	1241.78				
BIC	52.82				

**Table 6:** Parameters of the fitting of the adsorption isotherms of Langmuir, Freundlich, Temkin, Sips, and Redlich–Peterson models to the experimental data of the adsorption of Pb(II) on nano-TiO_2_.

Langmuir		Freundlich		Temkin	
q_e_ exp (mg g^−1^)	47.5	k_F_ ((mg g^−1^)/(mg L^−1^)^1/n^)	87.16 (1)	K_T_ (L g^−1^)	1049.45 (7)
q_m_ (mg g^−1^)	65.99 (6)	1/n	0.24 (5)	B_T_ (J mol^−1^)	219.85 (4)
k_L_ (L mg^−1^)	56.91 (2)	n	3.45 (7)		
R^2^	0.84	0.69		0.83	
RSS	577.34	577.34		1260.61	
BIC	48.36	77.25		56.95	
**Sips**		**Redlich-Peterson**			
$q_{m_{s}}$(mg g^−1^)	69.22 (1)	A (L g^−1^)	4191.41 (3)		
$k_{s}\;{\left(L{mg}^{-1}\right)}^{m_{s}}$	50.71 (3)	B (L mg^−1^)	60.75 (3)		
m_s_	0.85 (5)	β	0.97 (2)		
R^2^	0.82	0.82			
RSS	570.48	575.87			
BIC	48.23	50.73			

## Data Availability

The original data related to this research can be obtained at any time via the corresponding author’s email: juan.ramos5@unmsm.edu.pe.

## Supplementary Materials

The following supporting information can be downloaded at: https://www.mdpi.com/article/10.3390/molecules28124596/s1, Supporting section: Theoretical background of kinetic and isotherm adsorption models. Figure S1: (a,f) SEM images, (b,g) EDS mapping images, and (c,d,e,h,i,j) elemental EDS images for nano-TiO_2_ with C_0_ (Pb(II)) = 1.16 mg L^−1^. Figure S2: EDS spectrum for both nano-TiO_2_ samples exposed to two different Pb(II) concentrations. Figure S3: (a) TEM image of TiO_2_-PbC2 NPs (bar length of 100 nm), (b) PSD for nano-TiO_2_-PbC2, (c) Zoomed TEM image of nano-TiO_2_-PbC2, (d) SAED pattern of nano-TiO_2_-PbC2, and (e) crystallite size distribution histogram for nano-TiO_2_-PbC2 obtained from Rietveld refinement. C_0_ (Pb(II)) = 1.16 mg L^−1^. Figure S4: Linear models of Pb (II) adsorption kinetics on nano-TiO_2_. Adsorbent dose of 1 g L^−1^, pH 5.5. C_o_ = 47.54 mg L^−1^. Table S1: Fit parameters of linear models of adsorption kinetics of PFO, PSO, E, and IDM. References [79,80,81,82,83,84,85,86,87,88,89,90] are cited in Supplementary Materials.
