# Supplementary material for: Pb(II) Uptake from Polluted Irrigation Water Using Anatase TiO2 Nanoadsorbent
Source: Molecules. 2023 Jun 7;28(12):4596. doi: 10.3390/molecules28124596 (PMC10305539; doi:10.3390/molecules28124596)
Supplement: Supplementary file 1 [file molecules-28-04596-s001.zip › molecules-2403549-supplementary.pdf]

Supporting section

Theoretical background of kinetic and isotherm adsorption models

S.1. Kinetic adsorption models

S.1.1. Pseudo-first order (PFO) kinetic model

A widely used model to comprehend the adsorption kinetics of analytes onto solid adsorbent surfaces is the PSO kinetic adsorption model [RS1]. The adsorption rate is inversely proportional to the solute concentration in the solution and the number of open sites on the adsorbent surface, according to this basic kinetic model, Equation (S1) expresses this concept mathematically:

$$q_t = q_e(1 - e^{-k_1 t}) \quad \text{(S1)}$$

where q_t is the adsorbed amount with time (h) and q_e depicts the adsorbed amount (mg g^{-1}) at the equilibrium time t (h), and k_1 is the rate constant (h^{-1}), respectively [RS2].

S.1.2. Pseudo-second order kinetic (PSO) model

According to the PSO model, the rate of adsorption is inversely proportional to the square of the number of unoccupied sites on the adsorbent surface. The Equation (S2) describes the PSO model as follows:

$$q_t = \frac{q_e^2 k_2 t}{1 + q_e k_2 t} \quad \text{(S2)}$$

where k_2 (g mg^{-1} h^{-1}) is the PSO rate constant [RS3].

According to the PSO model, the adsorption rate at the initial stage, h (mg g^{-1} h^{-1}), at t = 0, is given by Equation (S3) [RS4]:

$$h = k_2 q_e^2 \quad \text{(S3)}$$

S.1.3. Elovich (E) kinetic model

The nonlinear E model describes the initial adsorption rate and the number of adsorption sites, that is represented by Equation (S4) [RS5]:

$$q_t = \frac{1}{\beta}\ln(\alpha\beta t + 1) \quad \text{(S4)}$$

where α (mg h^{-1}) is the initial adsorption rate and β (g mg^{-1}) is the desorption constant.

S.1.4. Intraparticle diffusion model (IDM)

The IDM relates the diffusion process of adsorbate molecules into the adsorbent's pore. expressed by Equation (S5) [RS6]:

$$q_t = k_p t^{\frac{1}{2}} + C_1 \quad \text{(S5)}$$

where C_1 (mg g^{-1}) is the intercept, which express the boundary layer effect and/or surface adsorption and k_p (mg g^{-1} $h^{-0.5}$) is the intraparticle diffusion rate constant.

S.2. Isotherm adsorption models

S.2.1. Langmuir isotherm model

Theoretically represents the adsorption process on homogeneous surfaces with a finite number of identical and non-interacting adsorption sites. The Langmuir formulation relates equilibrium adsorbate's amount, q_e (mg g^{-1}), that is adsorbed on the surface at a constant temperature, as given by Equation (S6):

$$q_e = \frac{q_m\, k_L\, C_e}{1 + k_L C_e} \quad \text{(S6)}$$

q_m (mg g⁻¹) is the maximum adsorption capacity of adsorbate, k_L (L mg⁻¹) defines the equilibrium constant known as the Langmuir adsorption energy, and C_e (mg L⁻¹) represents the adsorbate concentration at the equilibrium [RS7].

S.2.2. Freundlich isotherm model

An empirical model that reflects the solute's adsorption behavior onto a solid surface. It is extensively used in the field of adsorption and was proposed by Herbert Freundlich in 1906, and it is algebraically represented by Equation (S7) [RS8]:

$$q_e = k_F C_e^{\frac{1}{n}} \quad \text{(S7)}$$

where k_F ((mg g^{-1})/(mg L^{-1})$^{1/n}$) is the Freundlich's constant and n is defined as the heterogeneity of the adsorption sites.

S.2.3. Temkin isotherm model

This model is a semi-empirical model for describing molecule adsorption on solid surfaces. Moreover, the adsorption energy is inversely proportional to the square root of the coverage, and the heat of adsorption decreases linearly with it. The Temkin isotherm is expressed by the following Equation (S8) [RS9]:

$$q_e = \frac{RT}{B_T} \ln(K_T C_e) \quad \text{(S8)}$$

where K_T (L g^{-1}) is the Temkin isotherm constant, B_T (J mol^{-1}) is the adsorption constant, R is the gas constant (8.314 J mol^{-1} K^{-1}), and T (K) is the temperature.

S.2.4. Sips isotherm model

A model with three-parameter modification of the Langmuir isotherm, that is represented by the Equation (S9) [RS10]:

$$q_e = \frac{q_{m_s} k_s C_e^{m_s}}{1 + k_s C_e^{m_s}} \quad \text{(S9)}$$

where q_{m_s} (mg g^{-1}) is the Sips maximum adsorption capacity, k_s (L mg^{-1})^{m_s} is the Sips equilibrium constant, and m_s is the Sips's exponent.

S.2.5. Redlich–Peterson isotherm model

The process of a solute adhering to a solid surface, according to the Redlich-Peterson isotherm model, involves numerous layers and energetically heterogeneous adsorption sites. The model also assumes that at low concentrations, Langmuir's monolayer adsorption theory is followed. Redlich–Peterson isotherm is given by the Equation (S10) [RS11]:

$$q_e = \frac{AC_e}{1 + BC_e^{\beta}} \quad \text{(S10)}$$

where A (L g^{-1}), B (L mg^{-1}), and β exponent are empirical coefficients.

S.3. Statistical methods in model selection

S.3.1. Residual Sum of Squares (RSS)

RSS computes the difference between expected and actual values of the dependent variable. The goal is to narrow this gap; consequently, a lower RSS implies a better match between the model and the data. Equation (S11) represents the RSS formula:

$$RSS = \sum (y_i - \hat{y}_i)^2 \quad (S11)$$

where y_i and $\hat{y}_i$ are the actual and predicted values of the dependent variable [RS12].

S.3.2. Bayesian Information Criterion (BIC)

The BIC criterion was used to compare the best kinetic and isotherm models that describe the adsorption data for our adsorbent [RS12], it is mathematically expressed by Equation (S12):

$$BIC = n \ln\left(\frac{RSS}{n}\right) + k \ln(n) \quad (S12)$$

where n are the experimental points and k is the number of parameters in the model.

Supplementary Figures

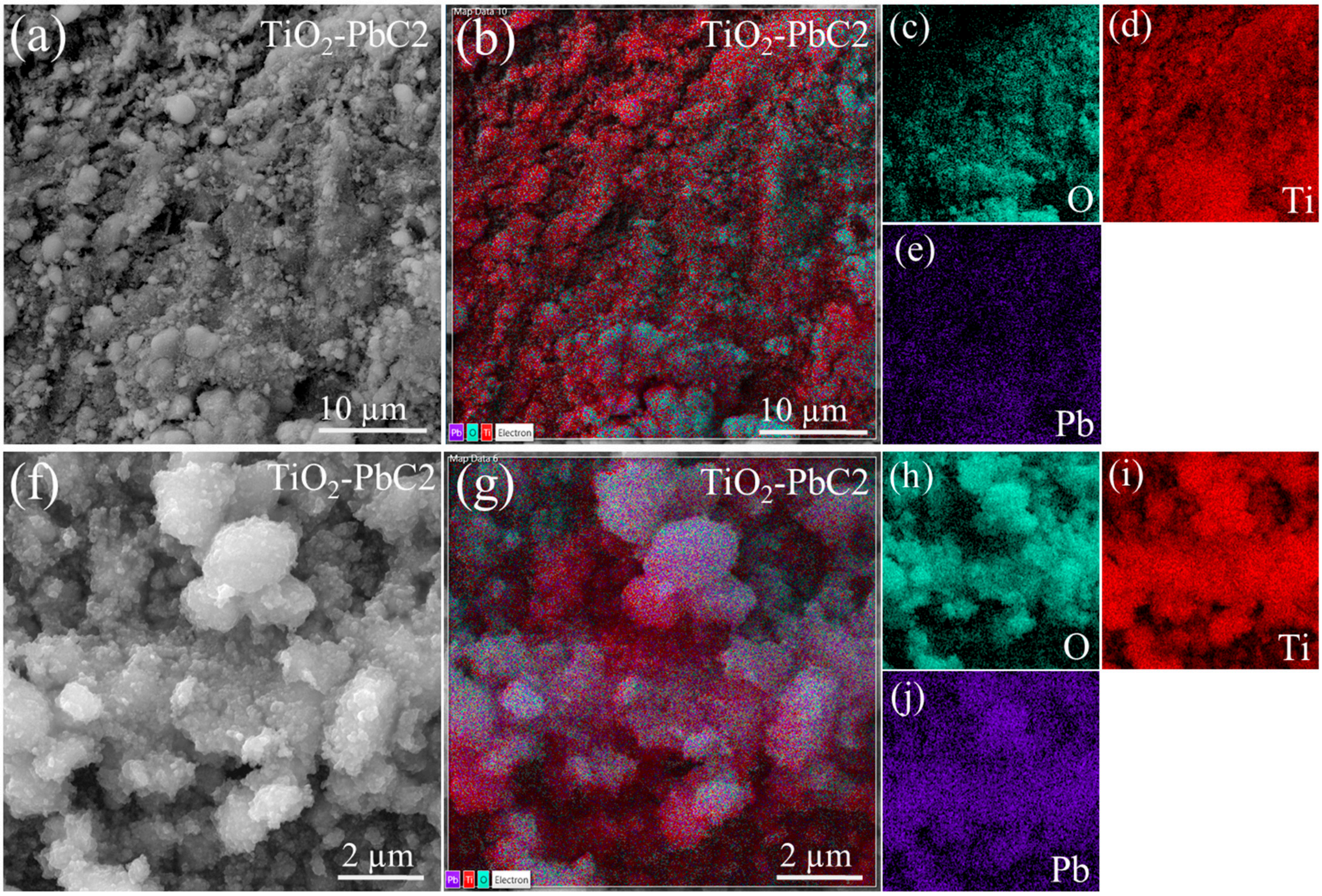

Figure S1: (a,f) SEM images, (b,g) EDS mapping images, and (c,d,e,h,i,j) elemental EDS images for nano-TiO_2 with C_0 (Pb(II)) = 1.16 mg L^{-1}.

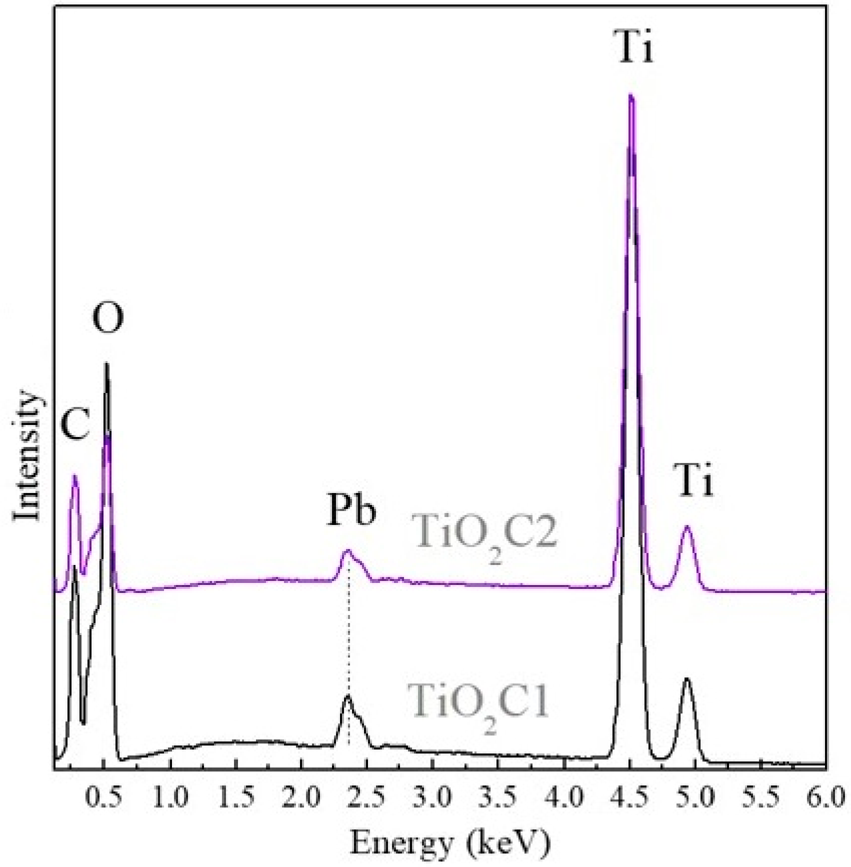

Figure S2: EDS spectrum for both nano-TiO_2 samples exposed to two different Pb(II) concentrations.

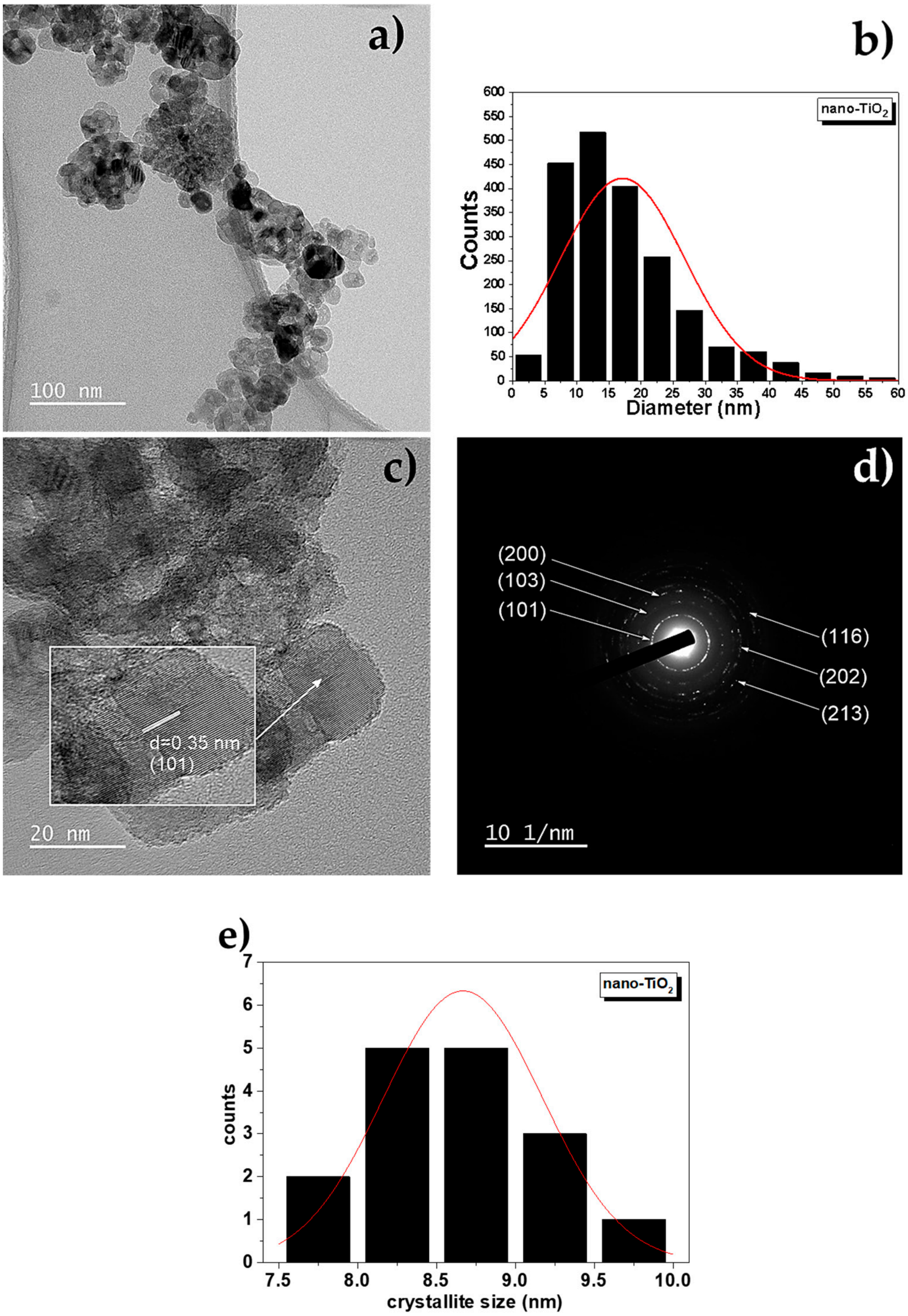

Figure S3: a) TEM image of TiO_2-PbC2 NPs (bar length of 100 nm), b) PSD for nano-TiO_2-PbC2, c) Zoomed TEM image of nano-TiO_2-PbC2, d) SAED pattern of nano-TiO_2-PbC2, and e) crystallite size distribution histogram for nano-TiO_2-PbC2 obtained from Rietveld refinement. C_0 (Pb(II)) = 1.16 mg L^{-1}.

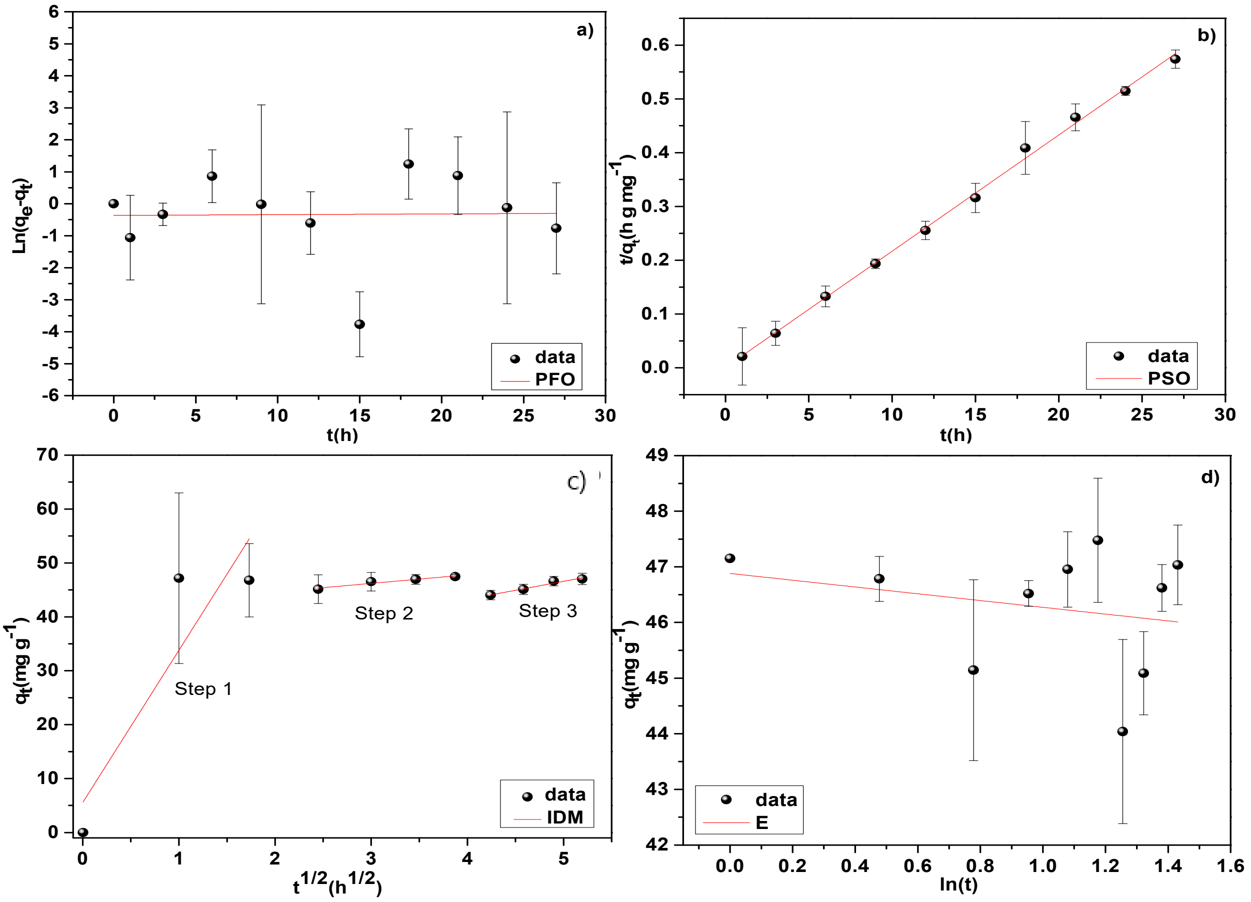

Figure S4: Linear models of Pb (II) adsorption kinetics on nano-TiO_2. Adsorbent dose of 1 g L^{-1}, pH 5.5. C_o= 47.54 mg L^{-1}.

Supplementary Tables

Table S1: Fit parameters of linear models of adsorption kinetics of PFO, PSO, E, and IDM.

Kinetic models		Parameter	Linear model
		q_e exp (mg g^{-1})	47.15
PFO		q_e (mg g^{-1})	0.69
		k_1 (h^{-1})	0.001
		R^2	0.0002
		RSS	20849.53
PSO		q_e (mg g^{-1})	46.29
		k_2 (g mg^{-1} h^{-1})	0.58
		h (mg g^{-1} h^{-1})	1242.80
		R^2	0.99
		RSS	20849.53
E		β (g mg^{-1})	−1.64
		α (mg h^{-1})	-1.3×10^{-32}
		R^2	0.13
		RSS	21430.69
IDM	Step 1	k_p (mg g^{-1} $h^{-0.5}$)	28.24
		C_1 (mg g^{-1})	5.56

	R^2	0.63
	RSS	46048.28
Step 2	k_p (mg g^{-1} h$^{-0.5}$)	1.59
	C_1 (mg g^{-1})	41.43
	R^2	0.93
	RSS	1785.16
Step 3	k_p (mg g^{-1} h$^{-0.5}$)	3.32
	C_1 (mg g^{-1})	29.98
	R^2	0.95
	RSS	1356.78

Supplementary references

RS1. Ho, Y.S. Citation review of Lagergren kinetic rate equation on adsorption reactions. *Scientometrics* **2004**, *59*, 171–177. 10.1023/B:SCIE.0000013305.99473.cf.

RS2. Tseng, R.L.; Wu, F.C.; Juang, R.S. Characteristics and applications of the Lagergren's first-order equation for adsorption kinetics. *J Taiwan Inst Chem Eng* **2010**, *41*, 661–669. 10.1016/j.jtice.2010.01.014.

RS3. Ho, Y.S.; McKay, G. Pseudo-second order model for sorption processes. *Process Biochemistry* **1999**, 34, 451–465. 10.1016/S0032-9592(98)00112-5.

RS4. Hamidpour, M.; Hosseini, N.; Mozafari, V.; Heshmati, M. Removal of Cd(II) and Pb(II) from aqueous solutions by pistachio hull waste. *Rev. Int. de Contam.* **2018**, *34*, 307–316. 10.20937/RICA.2018.34.02.11.

RS5. Wu, F.C.; Tseng, R.L.; Juang, R.S. Characteristics of Elovich equation used for the analysis of adsorption kinetics in dye-chitosan systems. *Chem. Eng. J.* **2009**, *150*, 366–373. 10.1016/j.cej.2009.01.014.

RS6. Wang, J.; Guo, X. Review on the Intraparticle Diffusion Adsorption Kinetics Model: Interpretation, Solving Methods and Applications. *SSRN* **2022**. 10.2139/ssrn.4120203.

RS7. Langmuir, I. The adsorption of gases on plane surfaces of glass, mica and platinum. *J Am Chem Soc* **1918**, *40*. 10.1021/ja02242a004.

RS8. Skopp, J. Derivation of the Freundlich Adsorption Isotherm from Kinetics. *J Chem Educ* **2009**, *86*, 1341–1343. 10.1021/ed086p1341.

RS9. Chu, K.H. Revisiting the Temkin Isotherm: Dimensional Inconsistency and Approximate Forms. *Ind Eng Chem Res* **2021**, *60*, 13140–13147. 10.1021/acs.iecr.1c01788

RS10. Sips, R. On the structure of a catalyst surface. *J Chem Phys* **1948**, *16*, 490–495. 10.1063/1.1746922.

RS11. Redlich, O.; Peterson, D.L. A useful adsorption isotherm. *J. Phys. Chem.* **1959**, *63*. 10.1021/j150576a611.

RS12. James, G.; Witten, D.; Hastie, T.; Tibshirani, R. An Introduction to Statistical Learning. **2017**. 10.1007/978-1-0716-1418-1.
